# Culinary Medicine Cooking Workshops as Add-On Therapy for Inpatients with Depression and Eating Disorders

**DOI:** 10.3390/nu16223973

**Published:** 2024-11-20

**Authors:** Sabrina Mörkl, Attila Varnagy, Jolana Wagner-Skacel, Theresa Lahousen, Daniel Brodtrager, Karl Sallmutter, Susanne A. Bengesser, Annamaria Painold, Martin Narrath, Lisa Pieter, Mary I. Butler, Annabel Mueller-Stierlin, Eva Z. Reininghaus, Sonja Lackner, Sandra Holasek

**Affiliations:** 1 Division of Medical Psychology, Psychosomatics and Psychotherapy, Medical University of Graz, 8036 Graz, Austria; sabrina.moerkl@medunigraz.at (S.M.); varnagy@varnagy.at (A.V.); jolana.wagner-skacel@medunigraz.at (J.W.-S.); 2 Division of Psychiatry and Psychotherapeutic Medicine, Medical University of Graz, 8036 Graz, Austria; theresa.lahousen@medunigraz.at (T.L.); daniel.brodtrager@uniklinikum.kages.at (D.B.); karl.sallmutter@uniklinikum.kages.at (K.S.); susanne.bengesser@medunigraz.at (S.A.B.); annamaria.painold@medunigraz.at (A.P.); martin.narrath@stud.medunigraz.at (M.N.); lisa.pieter@uniklinikum.kages.at (L.P.); eva.reininghaus@medunigraz.at (E.Z.R.); 3 Department of Agriculture, Ecotrophology and Landscape Development, Hochschule Anhalt, University of Applied Sciences, 06406 Bernburg, Germany; 4 Department of Psychiatry and Neurobehavioural Science, University College Cork, T12 K8AF Cork, Ireland; mary.butler@ucc.ie; 5 Institute for Epidemiology and Medical Biometry, Ulm University, 89075 Ulm, Germany; annabel.mueller-stierlin@uni-ulm.de; 6 Division of Immunology, Otto Loewi Research Center, Medical University of Graz, 8010 Graz, Austria; sandra.holasek@medunigraz.at

**Keywords:** culinary medicine, cooking workshops, depression, eating disorder, anorexia nervosa, nutritional psychiatry

## Abstract

Background: Culinary medicine integrates healthy eating with positive food experiences, offering a holistic approach to treating mental health disorders, such as depression and eating disorders, where disruptions in eating habits and mood are common. While traditional psychiatric treatments focus on medication and psychotherapy, culinary workshops provide a novel intervention for inpatient care. This study evaluated the effectiveness of culinary medicine cooking workshops as a supplementary treatment for psychiatric inpatients with depression and eating disorders. Methods: We assessed the feasibility of five cooking workshops led by a professional chef and nutritional therapist in 39 psychiatric inpatients (depression, *n* = 29; eating disorders, *n* = 10). Participants completed questionnaires on dietary habits, mood, and workshop feedback before and after the intervention. Results: The workshops were highly accepted, with 90% of participants reporting they would recommend them for recovery. Significant improvements were observed in mood (*p* < 0.001), sadness (*p* < 0.001), hopelessness (*p* = 0.002), and tiredness (*p* = 0.003) across the overall group. Patients with depression showed improvements in nearly all mood subscales, while those with eating disorders improved in sadness (*p* = 0.029). Conclusions: Culinary medicine workshops are a promising tool for enhancing mood and reducing hopelessness and tiredness in inpatients with depression. They also promote sustainable lifestyle changes that may benefit long-term physical and mental health. Future studies should explore the long-term impact of these interventions on psychiatric disorders.

## 1. Introduction

Depression and eating disorders are among the most chronic and disabling conditions worldwide and many patients do not respond to psychopharmacological treatment options in the acute setting [1]. Therefore, research on feasible add-on strategies for the inpatient setting is essential to improve treatment efficacy and outcomes. Nutrition has been at the center of medicine for thousands of years (e.g., in Chinese medicine and ancient Greece). Recent meta-analyses have shown that dietary improvement has a significant impact on psychiatric symptoms [2,3]. Specifically, dietary modifications have been shown to influence depressive symptoms such as anhedonia, loss of interest and drive, as well as anxiety and cognitive function [2,3,4]. However, nutrition-specific content still plays a subordinate role in medical training and therapy planning [5,6].

Changes in traditional diet (less natural, seasonal, regional) are associated with increased incidence of depression and other mental illnesses [7]. Fast-paced lifestyles have contributed to a loss of simple, healthy cooking skills [8,9,10]. Time pressure and workload have increased the demand for fast food, with a parallel decrease in the consumption of home-cooked and healthy meals [10,11,12]. In response, there has been a growth in the marketing of convenience foods that are energy-dense, low in nutrients, and high in salt, fat, and sugar [13,14]. These are the attributes of a so-called Western Diet, which was found to be associated with many mental and physical illnesses [15,16,17]. In contrast, health-promoting dietary recommendations are often summarized in the literature under the term “Mediterranean Diet” and are also represented in the Austrian dietary recommendations [18]. Essential cornerstones of these diets are a high proportion of plant-based foods, a diverse selection of vegetables, fruits, whole grain cereals, high-quality vegetable oils (such as olive oil), nuts, and seeds [19,20]. Likewise, additional fermented foods are recommended in the so-called “psychobiotic diet”, specially designed to target mental health [20].

The discipline of Culinary Medicine has emerged in recent years but has not yet found a place in clinical practice [21]. Practical skills for preparing health-promoting and palatable meals, along with implementation skills and nutrition knowledge, are key domains of food literacy [22]. Culinary medicine has the aim of providing knowledge on proper food and its preparation but is also dedicated to food enjoyment and the pleasure of eating. Preparing and eating meals together has several therapeutically valuable facets beyond simply teaching cooking skills. Cooking workshops act on several levels such as the teaching of theoretical and practical knowledge and skills, social aspects (cooking and eating together), and psychotherapeutic aspects (stopping ruminating negative thoughts, mindfulness, confrontation with food for eating disorder patients, self-care). The regular routine and shared meals could also positively influence patients’ mood in the short term and generate interest in diets that support the psyche, such as the Mediterranean diet. For example, in a study of 657 healthy adults, a 7-week cooking program significantly improved subjective mental and general health [23].

Although cooking interventions are used in outpatient therapeutic and rehabilitative settings, especially in occupational therapy [24], little is known about the feasibility of these interventions in an acute psychiatry setting and culinary medicine interventions have not yet been performed in patients with severe depression and eating disorders. Studies have shown that patients with depression and eating disorders display altered beliefs, thoughts, feelings, and behaviors concerning food [25,26] and may therefore respond differently to culinary medicine interventions. Knowledge on acceptance of cooking workshops in inpatients is therefore relevant for the improvement of inpatient therapy.

In this pilot project, we aimed to determine for the first time whether cooking workshops are feasible and accepted as a supplementary therapy in an acute psychiatry inpatient setting at the Medical University of Graz, Austria, and to what extent they are accepted by patients with depression and eating disorders as an addition to treatment as usual therapy. We hypothesize significant differences in the subjective acceptability of the workshops between the diagnostic groups.

## 2. Materials and Methods

### 2.1. Recruitment of Participants and Study Site

Inpatients between 18 and 80 years were recruited at the University Hospital for Psychiatry and Psychotherapeutic Medicine in Graz, Austria. Participants were included to reflect the broad demographic affected by depression and eating disorders. This age range allowed us to capture therapeutic benefits across adulthood, from younger adults, who may benefit from establishing healthy eating practices to older adults, who may experience improvements in mood, social engagement, and nutrition. The range also ensures practical feasibility, with participants being capable of independent participation in cooking workshops. The lower age limit (18 years) ensured participants were legally able to consent and possess the necessary independence for cooking activities, while the upper limit (80 years) considered potential physical limitations, yet still included older adults who stand to benefit from improved dietary habits and the social and therapeutic aspects of cooking.

Patients meeting the inclusion criteria were identified during routine treatment and ward rounds. Participation was voluntary. This study was approved by the Ethics Committee of the Medical University of Graz (protocol no: 34-387 ex 21/22) and conducted following the ICH Guidelines on Good Clinical Practice and the Declaration of Helsinki. Participants provided written informed consent. This pilot study is the first to test the acceptability and feasibility of cooking workshops in an inpatient setting with patients with depression and eating disorders. As this study was conducted during the pandemic (May 2022–November 2022), we decided to include a maximum of 8 persons per cooking workshop because of room size and planned to include at least 30 patients.

To be eligible for the study, the participants had to meet the following inclusion criteria: inpatients capable of giving consent between the ages of 18 and 80 years; diagnosis of depression or an eating disorder according to ICD-10. Exclusion criteria were as follows: neurological and psychiatric disorders other than those listed above, head injury, trauma, history of alcohol or drug abuse, acute suicidality, severe food allergies, acute gastrointestinal complaints, and patients with special dietary requirements (e.g., gluten-free, lactose-free, vegan). All participants received standard pharmacotherapy for depression or eating disorders throughout the study, with no adjustments made due to the cooking workshop intervention. Inclusion criteria were based solely on the primary diagnosis of depression or an eating disorder, without consideration of specific pharmacological treatments or individual responses to therapy. This approach allowed us to assess the impact of the cooking intervention as an adjunct to usual care.

### 2.2. Cooking Workshops

Cooking workshops took place every first week of the month in the occupational therapy area. The cooking workshops were conducted by a professional chef who was also a nutrition therapy student (AV), allowing for an integrated approach that combined culinary skills with nutritional guidance. This dual role ensured consistent methods and messaging throughout the workshops. Additionally, a multidisciplinary team contributed specialized expertise: A psychiatrist with a focus on nutritional medicine (SM) oversaw patient safety and therapeutic integration; occupational therapists (DB, KS) supported skill development and engagement; and a nutritionist (SL) helped design a workshop content to align with patients’ dietary needs. This collaborative approach provided a comprehensive, therapeutic experience for participants.

Recipes used in the workshops were easy to prepare and met the recommended standards of the Austrian food pyramid, the Mediterranean diet, and the psychobiotic diet [27], for the treatment of depressive symptoms. All created recipes were reviewed, discussed, and improved together with other co-authors of this study who added a psychiatric, therapeutic, and nutritional science perspective.

Recipes used were selected based on specific criteria to ensure they were easy to understand and prepared for psychiatric patients. Each recipe featured a limited number of familiar local ingredients, clear step-by-step instructions, and a short preparation and cooking time to maintain engagement and reduce complexity. During the workshops, the chef/nutrition therapy student (AV) provided hands-on, sequential guidance, supported by occupational therapists to assist as needed, ensuring that all participants could follow and complete each recipe confidently. The nutritional values were calculated with DGExpert v1.9 (Deutsche Gesellschaft für Ernährung, 2018).

The cooking workshops were structured in an interactive style to arouse interest, promote nutritional modification, and improve self-efficacy and cooking skills. Both patients with eating disorders and patients with depression took part in the same workshop group. The workshop lasted for approximately three hours. Every participant took part in one workshop during the inpatient stay. A short and low-threshold lecture on the connection between food and mental health was given before the workshop. During the cooking workshop, questions could be asked and information about ingredients and the way of preparation was provided. After the participants were introduced to hygiene basics, they were invited to actively contribute to the cooking procedure including peeling and chopping of food. In addition, the participants were offered to try different herbs and spices and were instructed to perceive the food with all senses. After the workshop, participants were invited to eat together at a shared table. No remuneration for the study participants was foreseen. Food was provided free of charge.

### 2.3. Questionnaires

Demographic data were collected including age and sex. Weight and height were used to calculate body mass index (kg/m^2^). Information concerning medical and psychiatric history was obtained. Before and after the workshop, participants completed self-report questionnaires on a variety of parameters including nutritional literacy and mood status. After the workshop, they were invited to answer a questionnaire concerning acceptance of the cooking workshop. To assess short-term changes in mood, the German version of the Profile of Mood States (Aktuelle Stimmungsskala, POMS) [28] was used. In order to characterize the eating habits of the study population, Mediterranean diet adherence was determined with the Mediterranean diet score (MDS) [29]. Additionally, the fat and fiber behavior questionnaire was used [30].

Nutritional literacy and specific questions on the acceptance of the cooking workshops were included in a modified version of the nutritional psychiatry questionnaire (NPQ) already published by our group [5] containing 29 questions. An English translation of the questionnaire can be found in Supplementary File S1. All questionnaires took about 10–15 min to complete.

### 2.4. Statistical Analysis

All statistical analyses were conducted using SPSS version 25 (SPSS, Inc., Chicago, IL, USA). Descriptive statistics, including means (M), standard deviations (SD), and medians (Mdn), were calculated for key demographic and study variables. The normality of continuous variables was assessed using the Shapiro–Wilk test. For comparisons between groups (e.g., participants with eating disorders vs. participants with depression), the unpaired t-test was used for normally distributed variables, and the Mann–Whitney U test was applied for non-normally distributed variables. For within-group comparisons of mood scores before and after the cooking workshop, paired t-tests were used for normally distributed data, and the non-parametric Wilcoxon signed-rank test was applied when the data did not meet normality assumptions. This analysis was conducted both for the entire sample and separately for each subgroup (depression and eating disorders). All tests were two-tailed, with statistical significance set at *p* = 0.05.

## 3. Results

### 3.1. Participants

Sociodemographic Data and Medical History

A total of 45 inpatients at the University Hospital for Psychiatry and Psychotherapeutic Medicine in Graz took part. Of the 45 participants, 39 returned the questionnaires, which corresponds to a response rate of 87%.

About 29 (74%) patients were diagnosed with a depressive episode and 10 (26%) patients with an eating disorder (anorexia nervosa *n* = 6; bulimia nervosa *n* = 4). The mean age was 41.43 (SD = 13.44) years. The participants’ age ranged from 19 to 64 years. Of the participants, 30 (77%) were women and 9 (23%) were men. All participants in the eating disorder group (*n* = 10) were female.

For depressed patients, the mean weight was 74.48 kg (SD = 15.96), and the mean BMI was 25.37 kg/m^2^ (SD = 4.06). The weight ranged from 54 kg to 112 kg. The mean weight in patients with eating disorders (*n* = 10) was 45.78 kg (SD = 2.97), the mean BMI was 16.84 kg/m^2^ (SD = 2.97), and the weight ranged from 32 to 54 kg.

The baseline characteristics of participants, including age, weight, height, and body mass index (BMI), are shown in Table 1.

### 3.2. Acceptability

Questions About the Cooking Workshop

When asked about their general impression of the cooking workshop on a scale from 1–10, 72% (*n* = 28) of patients rated it “very good” (10/10 points), 9 (23%) patients rated between 7 and 9 points, and 1 patient (2.5%) rated 2 points.

Thirty-five (90%) patients indicated that they would definitely like to attend another cooking workshop. Three (8%) patients said that they may want to attend another cooking workshop and 1 patient (with anorexia nervosa) (2%) did not want to attend cooking workshops again in the future. Thirty-seven (95%) participants stated that they would definitely recommend such a cooking workshop to others and two patients (one with an eating disorder and one with depression) stated that they would not.

About 22 (57%) patients considered the cooking workshop to be very useful, 16 (41%) to be rather useful (6 to 9 points), and 1 (2%) patient did not consider it useful for the additional treatment of mental illness.

When asked what could be improved, most of the patients with depression were content with the current design of the workshops. Two patients said that they wanted to participate even more actively. A patient mentioned that he wanted the workshops to last longer, as three hours was not enough, and has regular cooking workshops integrated into therapy. It was also recommended by one patient to provide a handout with the most important take-home messages from the workshop. Patients with eating disorders clearly seemed to struggle with the workshops. For example, two of them found the workshops too complex and the offered variety of foods challenging (“*it was too much for me and too colorful*”).

### 3.3. Changes in Profile of Mood States (POMS)

In terms of general mood measured by POMS total score, a significant improvement could be found in the whole sample (*t*(38) = 4.20, *p* < 0.001) and also for the depression subgroup (*t*(28) = 4.26, *p* < 0.001). However, there was no significant improvement in the eating disorders subgroup (*t*(9) = 1.26, *p* = 0.238).

**Figure 1:**
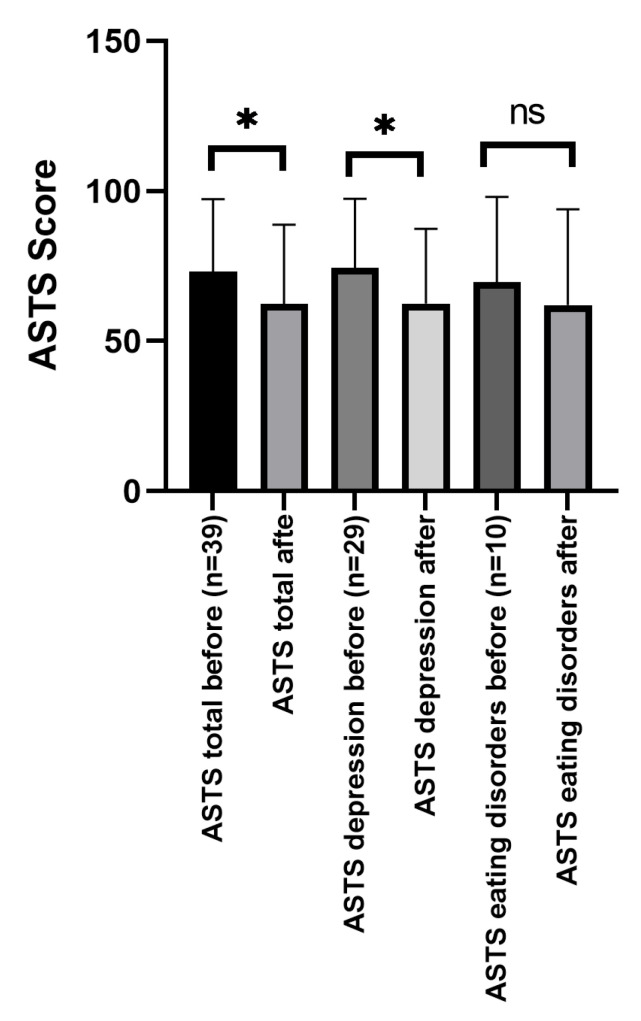
Changes in the total POMS score before and after participation in the cooking workshop. ASTS = Aktuelle Stimmungsskala–German Translation of Profile of Mood States (POMS). * *p* < 0.05, ns = non significant.

Figure 1 shows the change in the total POMS score of all participants (*n* = 39), depressive participants (*n* = 29), and participants with eating disorders (*n* = 10) before and after participation in the cooking workshop.

Regarding the subcategories of the POMS, significant improvements were shown for depressed patients (*n* = 29) regarding sadness (*t*(28) = 4.14, *p* < 0.001), hopelessness (*t*(28) = 3.58, *p* = 0.001), fatigue (*t*(28) = 3.13, *p* = 0.004), positive mood (*t*(28) = 2.55, *p* = 0.016) but not for anger (*t*(28) = 1.35, *p* = 0.186). For patients with eating disorders, significant improvements could only be found for sadness (*t*(9) = 2.59, *p* = 0.029) before and after the workshop but not in the other subcategories. Table 2 gives an overview of group differences for POMS total score and subscores before and after the workshop.

### 3.4. Data on Dietary Patterns, Nutritional Habits, Nutrition Knowledge, Nutritional Therapy

Mediterranean diet adherence and fat and fiber intake were determined via questionnaires before the workshop. Regarding the MDS, no significant differences could be found between the participants with depression (Mdn = 6) and the participants with eating disorders (Mdn = 7) (U = 197, *p* = 0.086). Nine (23%) participants reached 8 points or above indicating medium to high Mediterranean diet adherence. Regarding the fat and fiber questionnaire, there was a significant difference in fiber intake between the groups, whereas patients with eating disorders reported eating significantly more fiber (*t*(36) = −2.16, *p* = 0.037); however, there were no reported differences in fat intake. The lowest total fat and fiber score (1.40) and fat score (1.38) were reported by a patient with anorexia nervosa and the lowest fiber value (1.29) by a participant with depression.

About 23 (59%) patients reported usually eating in company, and 16 (41%) reported eating alone; 10 (26%) patients considered nutritional aspects to be very important, 27 (69%) rather important, and 2 (5%) rather not (4 and 5 points) important for mental health.

General knowledge of nutrition was rated with a mean score of 6.10 (SD 1.80) on a scale from 1 to 10. About 87.2% (*n* = 34) of patients stated that they would like to increase their knowledge of nutrition. Patients rated their current diet with an average of 5.43 points (SD 2.25) out of 10, whereas patients with depression gave themselves a rating of 5.58 (SD 2.13) and patients with eating disorders gave themselves a rating of 5.00 (SD 2.67).

On a scale from 1 to 10, participants rated the general diet in Austria with 4.46 (SD 1.82) points and the diet in Austria in people with mental illness with 3.74 (SD 1.72) points. Hospital food was rated with an average of 5.92 (SD 1.98) points. Of the respondents, only 8 (20.51%) have never tried dietary approaches to treat their mental illness;14 (36%) patients reported never, 13 (33%) rarely (2 to 5 points), and 7 (17%) to often talk with their therapists about nutrition. Only 18% (7) of patients considered their diet more often, 36% (14) of patients rarely, and 43% (18) of patients never considered their diet concerning their prescribed medication.

Twenty-five (64.10%) patients were taking nutritional supplements for their mental illness. Patients’ answers to the additional question “If yes, which ones?” varied widely, in some cases not only nutritional supplements but also herbal supplements and foods (such as kefir) were named. Table 3 lists the answers given.

However, 30 (77%) participants reported that supplements were never recommended to them by their psychiatrist/psychologist; 15.4% (*n* = 6) of participants stated that a special diet was recommended for them by their psychiatrist/psychologist/psychotherapist. Participants responded with the following free text answers: “diet according to Austrian food pyramid”, “ healthy and balanced diet”, “traditional Chinese medicine (TCM) diet”, “ayurveda diet”, “to cook more at home and avoid fast food”, and “interval fasting”.

#### Questions on Food Literacy and Cooking Skills

When asked to rate their practical kitchen skills, patients gave themselves a mean rating of 6.85 (SD 1.97) points on a scale from 1 to 10. Regarding cooking competency (“*I feel competent to prepare a wholesome meal for myself and others*”), patients rated themselves with a mean of 5.82 points (SD 2.98). There was no significant difference between the self-rating of practical skills and cooking competency between the two groups. Both patients with depression and eating disorders reported feeling too bad to cook for themselves and not having time for cooking. While patients with depression reported more problems concerning financial expenses, patients with eating disorders reported a lack of skills in the kitchen.

The need for nutritional training of psychiatrists/psychologists and psychotherapists was rated 8.31 (SD 1.94) on a scale from 1 (no need) to 10 (great need). Furthermore, patients rated 7.23 points on average (SD 1.97) when asked whether they would think this would improve their therapy. About 22 (56%) patients rated that there was a very great and 17 (44%) that there was a rather great (6 to 9 points) need for further research in the area of nutritional psychiatry.

## 4. Discussion

This study reports, for the first time, the acceptability of cooking workshop intervention for inpatients with depression and eating disorders. Cooking workshops were well-received and tolerated, especially in patients with depression, with 90% of participants stating that they would definitely attend another workshop. Overall, patients considered cooking workshops to be an important support for inpatient therapy. Furthermore, we found significant improvements in mood before and after the workshop in the whole group and the subgroup with depression. Depressed patients could significantly improve on nearly all subcategories of the POMS (such as sadness, hopelessness, fatigue, and positive mood). In patients with eating disorders, there was only a significant improvement in sadness but in no other subscale. No difference could be found in Mediterranean dietary adherence between the groups. General nutrition literacy ratings were 6/10 points on average and most of the patients (87.2%) wanted to increase their knowledge.

The evaluation of the NPQ shows that the majority of inpatients with depression and eating disorders enjoyed the cooking workshop and consider these tools to be an important addition to treatment as usual. Cooking workshops were well-accepted by inpatients with depression and eating disorders. The evaluation of the NPQ questionnaires shows that more than 90% of the patients with depression enjoyed the cooking workshop and would attend it again. Patients with depression also consider the cooking workshop to be a useful addition to the treatment of mental illness. Our hypothesis that patients with eating disorders and depression would respond differently to the workshops was partly proven. For patients with depression, significant changes in mood could be found in the POMS scores as they may have perceived the cooking activity as distracting and relaxing, while for patients with eating disorders, it seemed rather confrontational and challenging.

Nearly 80% of the participants had already tried dietary approaches to treat their mental health condition; however, two-thirds never or rarely talked to their psychiatrists or therapists about nutrition. Over half of the patients (64.10%) were apparently self-medicated with nutritional supplements (see Table 3), as 77% reported supplements were never recommended to them by their psychiatrist or psychologist. Even on fewer occasions (in only 6 patients), a special diet was recommended by their psychiatrist or psychologist. This underlines the necessity of further training of psychiatrists, psychologists, and psychotherapists in the basics of nutrition [5], which is also wished for by the patients as they consider nutritional training for mental health professionals to be very important and believe it could improve therapy outcomes. Awareness of dietary factors in therapy could also be created by the use of special instruments such as the nutrimental screener, which prompts referral to a dietician if needed and also wanted by the patient [31].

Our questionnaire showed that nutrition is important to patients, but they often do not have the desire or strength to cook for themselves. While depressed patients stated bad mood, motivation, food insecurity, and time problems as the main reasons, patients with eating disorders named bad mood, time reasons, and a lack of skill in the kitchen. Food insecurity is an upcoming issue in psychiatry patients, a condition defined by limited or uncertain access to sufficient, nutritious food for a healthy life and a high sense of well-being. Studies suggest that food insecurity is associated with poor mental health outcomes, including depression and anxiety [32,33,34,35]. Huddleston-Casas et al. [35] reported a bidirectional relationship between food insecurity and depression in which the experience of being food insecure can cause depression and being depressed can contribute to food insecurity. Associations between food insecurity and mental health have aggravated during the COVID-19 pandemic [36]. They have the potential to increase mental health disparities over the long term.

This study has several strengths: It is one of the first studies testing the acceptability and feasibility of a culinary medicine-cooking workshop in an acute psychiatry setting for patients with depression and eating disorders. A multi-professional team (chef and nutritional therapy student (AV), a psychiatrist trained in nutritional medicine (SM), occupational therapists (DB, KS), and nutrition scientist (SL) delivered interventions. All cooked dishes were prepared together and created for the cooking workshops from both a nutritional and pleasure therapy point of view.

However, some limitations need to be addressed: First, the significance of this study could be limited due to the low sample size in both groups. As mentioned above, this was a pilot study testing a culinary medicine intervention for the first time in a psychiatric inpatient setting. Furthermore, the trial was conducted during the COVID-19 crisis with restrictions on the number of participants. In addition, as participation was voluntary, only patients with a primary interest in culinary interventions took part, which could affect subjective answers to the questionnaires. Furthermore, patients may have over- or underreported their current eating behavior and nutritional intake, as we could not find a significant difference in Mediterranean diet adherence in the groups. Especially patients with disturbed body perception often inaccurately report a healthier lifestyle [37,38] and in patients with eating disorders, over-reporting is quite common [39,40]. Patients with eating disorders reported more fiber intake than patients with depression, which is in line with other studies showing low fiber intake in this group [41,42]. Due to limited resources and the cross-sectional nature of the project, no dietary diaries were kept with patients. Furthermore, short-term improvements in mood could be found; however, our cross-sectional study design does not allow us to answer whether these interventions have any long-term effects. Furthermore, longitudinal studies should test the impact of cooking workshops on mood and dietary patterns. Standardized questionnaires were used for scientific comparability. Finally, the changing menus and the different degrees of interactions in the cooking sessions between the patients could have influenced mood in a positive or negative manner. Furthermore, gender-specific conclusions cannot be drawn due to the small sample size. As there is a higher prevalence of depression and eating disorders among women, they also participated more in cooking workshops. Whether there are gender differences regarding the impact of cooking workshops needs to be answered in larger studies.

Cooking workshops seem to be a feasible and acceptable intervention in an inpatient psychiatry setting for patients with depression and eating disorders. However, they should be studied longitudinally and become accessible to patients with a variety of disorders. One-time participation may not be sufficient to achieve long-lasting improvements. There should also be the possibility of attending further cooking workshops for patients who are willing to do so. Sharing a meal may improve both the social skills of patients and their access to nutrition. For example, it was shown in prior studies that people who eat socially feel happier and more trusting of others and that social eating is an important mechanism for social bonding [43,44]. It would be helpful for patients undergoing prolonged inpatient psychiatric treatment to have the opportunity to cook together and share meals, as eating alone increases the odds of depressive symptoms and suicidality [45]. Furthermore, the findings of this pilot project could also be taken into account in the design of hospital catering and re-instating the common meal in the dining room for all inpatients, which was not possible during the COVID-19 crisis.

## 5. Conclusions

Cooking workshops could be a feasible and acceptable add-on therapy in biopsychosocial inpatient treatment as they reinforce salutary factors [46]. More research is needed to determine how nutrition education programs lead to sustained knowledge and behavior change within this population.

## Figures and Tables

**Table 1:** Characteristics of participants in cooking workshops.

Characteristics of Participants	Participants with Eating Disorders (*n* = 10)	Participants with Depression (*n* = 29)	Total (*n* = 39)	*p*-Value
	M ± SD			
Age (years) (M ± SD)	36.8 ± 14.58	43.03 ± 12.91	41.43 ± 13.44	0.210 (*t*-test)
Weight (kg) (M ± SD)	45.78 ± 17.86	74.48 ± 15.96	66.93 ± 19.40	<0.001 *** (U-test)
Height (m) (M ± SD)	1.65 ± 0.05	1.70 ± 0.081	1.69 ± 0.078	0.450 (U-test)
BMI ^1^ (kg/m^2^) (M ± SD)	16.84 ± 2.97	25.37 ± 4.06	23.13 ± 5.35	<0.001 *** (U-test)
Female (*n*, %)	10, 100%	20, 68.96%	30, 77%	

^1^ BMI = body mass index, M = mean, SD = standard deviation.*** *p* < 0.001 (*p*-values calculated using unpaired *t*-test or Mann–Whitney U test as appropriate).

**Table 2:** Scores of POMS questionnaire before and after the cooking workshop.

POMS Scores, Before and After the Cooking Workshop	Participants with Eating Disorders (*n* = 10)	Participants with Depression (*n* = 29)	Total (*n* = 39)	Difference Between Groups (*p*-Value)
M ± SD				
Total score, before (M ± SD)	36.8 ± 14.58	43.03 ± 12.91	41.43 ± 13.44	0.601 (*t*-test)
Total score, after (M ± SD)	45.78 ± 17.86	74.48 ± 15.96	66.93 ± 19.40	0.950 (*t*-test)
Subscore sadness, before (M ± SD)	1.65 ± 0.05	1.70 ± 0.081	1.69 ± 0.078	0.417 (*t*-test)
Subscore sadness, after (M ± SD)	16.84 ± 2.97	25.37 ± 4.06	23.13 ± 5.35	0.669 (*t*-test)
Subscore hopelessness, before (M ± SD)	10.72 ± 5.98	8.30 ± 4.59	10.10 ± 5.70	0.252 (*t*-test)
Subscore hopelessness, after (M ± SD)	8.31 ± 5.31	7.50 ± 5.50	8.10 ± 5.30	0.682 (U-test)
Subscore fatigue, before (M ± SD)	16.10 ± 6.45	16.10 ± 8.07	16.10 ± 6.79	0.999 (*t*-test)
Subscore fatigue, after (M ± SD)	13.86 ± 7.02	14.00 ± 8.65	13.90 ± 7.35	0.960 (*t*-test)
Subscore positive mood, before (M ± SD)	27.00 ± 9.47	26.30 ± 10.33	26.82 ± 9.52	0.844 (*t*-test)
Subscore positive mood, after (M ± SD)	30.41 ± 7.35	28.00 ± 8.99	29.79 ± 7.77	0.405 (*t*-test)
Subscore anger, before (M ± SD)	4.86 ± 3.21	6.50 ± 3.83	5.28 ± 3.41	0.194 (U-test)
Subscore anger, after (M ± SD)	4.52 ± 2.98	6.10 ± 4.93	4.92 ± 3.58	0.233 (U-test)

M = mean, SD = standard deviation. (*p*-values calculated using unpaired *t*-test or Mann–Whitney U test as appropriate).

**Table 3:** Free text answers to the question: “Did you ever take supplements for your mental health? If yes, which ones”?

Supplements Taken by Patients with Eating Disorders	Supplements Taken by Patients with Depression
Vitamins, enzymes, and trace elements	Vitamins, enzymes, and trace elements:
Vitamin D3	Multivitamins
Vitamin B12	Vitamin B12
Vitamin B complex	Vitamin B complex
Magnesium	Iron
Potassium	Magnesium
Calcium	Vitamin D
Iron	Vitamin K2
	Coenzyme Q10
	Selenium
Herbal supplements:	Herbal supplements:
St. Johns Wort (Hypericum perforatum)	Cannabidiol
	Ashwagandha (Withania somnifera)
Special foods:	
Omega-3 oil	Special foods:
Maltodextrin	Probiotics
Sip food	Omega-3
	Kefir

## Data Availability

The data presented in this study are openly available in Zenodo at https://doi.org/10.5281/zenodo.14012780.

## Supplementary Materials

The following supporting information can be downloaded at https://www.mdpi.com/article/10.3390/nu16223973/s1, Supplementary File S1: English translation of the adapted version of the Nutritional Psychiatry Questionnaire (NPQ).
